# Automatic Detection of the Cyclic Alternating Pattern of Sleep and Diagnosis of Sleep-Related Pathologies Based on Cardiopulmonary Resonance Indices

**DOI:** 10.3390/s22062225

**Published:** 2022-03-14

**Authors:** Jiajia Cui, Zhipei Huang, Jiankang Wu

**Affiliations:** 1 University of Chinese Academy of Sciences, Beijing 101408, China; cuijiajia17@mails.ucas.ac.cn; 2 CAS Institute of Healthcare Technologies, Nanjing 210046, China; jkwu@ucas.ac.cn

**Keywords:** cyclic alternating pattern, cardiopulmonary resonance indices, sleep-related pathology, machine learning

## Abstract

The cyclic alternating pattern is the periodic electroencephalogram activity occurring during non-rapid eye movement sleep. It is a marker of sleep instability and is correlated with several sleep-related pathologies. Considering the connection between the human heart and brain, our study explores the feasibility of using cardiopulmonary features to automatically detect the cyclic alternating pattern of sleep and hence diagnose sleep-related pathologies. By statistically analyzing and comparing the cardiopulmonary characteristics of a healthy group and groups with sleep-related diseases, an automatic recognition scheme of the cyclic alternating pattern is proposed based on the cardiopulmonary resonance indices. Using the Hidden Markov and Random Forest, the scheme combines the variation and stability of measurements of the coupling state of the cardiopulmonary system during sleep. In this research, the F1 score of the sleep-wake classification reaches 92.0%. In terms of the cyclic alternating pattern, the average recognition rate of A-phase reaches 84.7% on the CAP Sleep Database of 108 cases of people. The F1 score of disease diagnosis is 87.8% for insomnia and 90.0% for narcolepsy.

## 1. Introduction

Sleep, which accounts for nearly a third of human life, is an important function that helps the body to recover. It has been proven that sleep could help to reduce stress, regulate hormone balance, stabilize appetite and cardiovascular function [1,2,3]. At the same time, sleep is essential for the recovery of the brain function, which is closely related to brain development, learning, memory and mental health of human beings [4]. A lack of sleep will cause different degrees of harm to the body and mind [5]. The monitoring of sleep and the detection of sleep-related diseases are of great significance in people’s daily life as well as in clinical treatment.

Sleep is known to rhythmically regulate autonomic nervous system activity. Quiet sleep is associated with increased parasympathetic arousal and activity, while rapid eye movement (REM) sleep is relevant to the increased sympathetic activity [6]. The sleep structure is based on the cyclical alternation of two main neurophysiological states: REM and NON-REM (NREM) sleep [7]. The alternations of non-REM and REM sleep constitute the sleep cycle, and its recurrence during the night determines the classical progressive sleep mode.

Many researchers suggest that both slow and fast electroencephalogram (EEG) activation complexes are involved in the structural organization of sleep [8]. Deep sleep is established and maintained through a process of periodic EEG instability accompanied by mild autonomic fluctuations in the wake state [9]. In contrast, the breakdown of slow-wave sleep and the introduction of REM sleep are mainly associated with EEG desynchronization and strong activation of muscle and autonomic nerve functions [10].

The Cyclic Alternating Pattern (CAP) is the periodic EEG activity occurring during NREM sleep. It is characterized by cyclic sequences of cerebral activation (phase A) followed by periods of deactivation (phase B) which separate two successive phase A periods with an interval <1 min [11]. Phase A period together with the following phase B period define a CAP cycle [12]. Detailed investigation has ascertained that the spontaneous EEG fluctuations of CAP are implicated in the subtle mechanisms that regulate the production and attenuation of slow-wave activities during sleep [13]. Different components of CAP have a sculpturing effect on the profile of the sleep cycle.

Phase A periods are subdivided into three subtypes. The abundance of A1 subtype in the descending branches and grooves may be an EEG expression of brain mechanisms involved in release activity, while the dominance of A2 and A3 subtypes in the ascending branches reflects the REM-on drive [14]. Therefore, in addition to a variety of EEG features, the activation complex also has non-random distribution characteristics at night, and has obvious periodicity in NREM sleep within the CAP framework, e.g., [15,16,17]. CAP is regarded as the main expression of sleep microstructure. CAP can be recognized in the sleep of both adults and children and it is a sensitive tool for studying sleep disorders throughout the life cycle [18,19].

Several efforts have been made to develop a reliable automatic CAP-scoring algorithm [20,21,22,23]. Most of these methods rely on the extraction of spectral features from the EEG and on the application of machine-learning algorithms, such as the k-nearest neighbor, support vector machine, artificial neural network, decision trees, and deep neural network [24,25]. However, EEG acquisition needs to be carried out under the guidance of experts, and the wearing of the equipment will also affect the sleep state of the subjects [26]. The preprocessing [27], recognition [28] and analysis [29] of EEG signal are complicated [30].

Some researchers and institutions have tried to classify sleep stages by physiological signals instead of EEG. As physiological signals that contain the physiological characteristics and autonomic nerve status information of the human body, Electrocardiograph (ECG), respiration and the three-axis acceleration signals on the chest have attracted much attention [31,32,33]. Wilhelm Daniel Scherz et al. used Heart Rate Variability (HRV) characteristics of three transformation domains to determine sleep stages [34]. Mourad Adnane et al. attempted to extract heart rate variability features from time domain, frequency domain, detrend fluctuation analysis and window detrend fluctuation analysis, and used a support vector machine to classify sleep and wake stage, with an average accuracy of 79.3% [35]. Martin Oswaldo Mendez et al. extracted HRV and body movement using a time-varying auto-regression model, and classified REM and NREM using hidden Markov model [36], and the time-varying relationship between EEG and HRV are studied [37]. Eline R.de Groot et al. researched on the value of cardiorespiratory parameters for sleep state classification in preterm infants [38].

Considering the connection between the heart and brain of people [39,40,41], the CAP, which derives from the transformation of EEG, affects the excitability of human nerves and then reflects not only in body movements and changes of heart rate, but also in respiration [42,43]. Cardiopulmonary characteristics of people show diverse manifestations in different stages of the sleep. Inspired by the causality analysis of the modulation of heart rate form respiration, cardiopulmonary resonance indices (CRI) are defined to measure the status of the cardiopulmonary resonance system by adopting the cardiopulmonary system circuit model based on the causality analysis in frequency domain [42].

In this research, we first expanded the index system of CRI, combined with the physiological significance of the cardiopulmonary coupling model. CRI in different sleep stages of different people were analyzed in detail in this paper. Inspired by the results of the statistical analysis of the cardiopulmonary characteristics of healthy people and patients with sleep-related diseases, we proposed an automatic recognition and disease diagnostic scheme of CAP based on CRI. The scheme combines the variation and stability of CRI using Hidden Markov [44] and Random Forest model [45]. In addition to the recognition of CAP, this scheme can also diagnose sleep-related diseases through the cardiopulmonary characteristics. Compared with the features of the directly acquired signals, such as ECG, three-axis acceleration signals, etc., the proposed method explores the deep rhythm in the cardiopulmonary system during human sleep, and is effective to conquer the weaknesses in signal acquisition and processing in clinical trials. At the same time, this study also proves the existence of the mind-brain connection in the human body.

In this paper, for materials and methods, Section 2 describes the database used in the study and the data preprocessing before calculating CRI. Section 3 describes the CRI and expands the index system, and statistical analysis of the cardiopulmonary characteristics of people with non-pathology and patients with sleep-related diseases, then gives the classification and recognition scheme. Section 3 and Section 4 give the details and effects of the methods. The last section is the conclusion and future work.

## 2. Materials and Methods

### 2.1. Data

This section describes the data set and the data processing in this research.

#### 2.1.1. Data Set

The experimental data used in this study is obtained from the CAP Sleep Database [46] in MIT-BIH database. The CAP Sleep Database is a collection of 108 polysomnographic recordings registered at the Sleep Disorders Center of the Ospedale Maggiore of Parma, Italy. The waveforms include at least three EEG channels, Electro-Oculogram (EOG) (two channels), Electromyography (EMG) of the submentalis muscle, bilateral anterior tibial EMG, respiration signals, the acceleration and gyroscope signals in the chest and ECG [47].

The database includes 108 cases of people with non-pathology and several sleep-related pathologies. Expert neurologists who trained at the Sleep Center provided the scoring of the sleep macrostructure, according to the Rechtschaffen & Kales rules [48], including SLEEP−REM, SLEEP−S0, SLEEP−S1, SLEEP−S2, SLEEP−S3, SLEEP−S4, while the CAP was detected in agreement with Terzano’s reference atlas of rules [49], including MCAP−A1, MCAP−A2 and MCAP−A3 in the NREM period every 30 s. Sleep stages S0, S1 and S2 are collectively called light sleep stage, while S3 and S4 are referred to as deep sleep stage.

**Figure 1:**
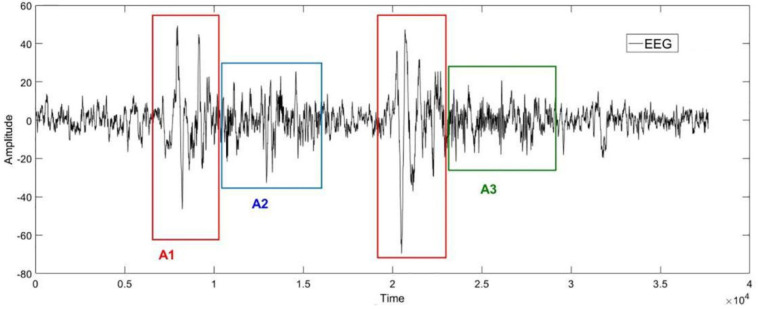
An example of the EEG in cyclic alternating pattern (CAP) in sleep stage 2. The horizontal axis represents the sampling point, and the vertical axis represents the amplitude of the signal. The shapes of the EEG signals in red, blue, and green boxes correspond to the CAP−A1, CAP−A2, and CAP−A3, respectively.

The Figure 1 below is an example of the EEG in sleep stage 2. Phases A1, A2 and A3 are framed in the diagram according to the labels from experts. As shown in Figure 1, the Phase A periods are subdivided into three subtypes [50]: Subtype A1: synchronized events with low impact on autonomic and somatomotor activities; Subtype A2: mixed synchronized–desynchronized EEG events with an intermediate influence on the autonomic and somatomotor activities; Subtype A3: predominantly desynchronized EEG events with heavy effects on the autonomic and somatomotor activities.

#### 2.1.2. Data Selection and Preprocessing

The ECG and respiration signals of people with non-pathology, insomnia and narcolepsy are used in this study. For the signals in the database, stationary signals are selected for analysis. 

Wavelet analysis is used to remove the baseline drift of the signal and a Pasteur band-pass filter is used to remove the noise. Then, for respiratory signals, we define the signal quality function as follows:

(1) $$Q=\frac{\sum_{0.03}^{0.5}psd(res)}{\sum psd(res)}$$

where *res* represents the respiratory signal, *psd* represents power spectral density function. *Q* value expresses the concentration degree of the power spectrum of respiratory signal, and the stability of respiratory frequency. Considering the respiratory signal is concentrated in the frequency domain from 0.03 Hz to 0.5 Hz [51], the signals which meet the requirement that *Q* > 0.85 are chosen [43,52]. This process ensures the quality of the respiratory signal to be representative of the breathing condition of the subject. 

As for the ECG, to ensure RR intervals (intervals between adjacent R waves) in the normal range and without a mutation because of premature beat or other physiological phenomena [53], we use interpolation as a substitute for points that do not meet the following conditions based on the Gaussian model [54]:

(2) $$\begin{array}{l} \left|RRI_{i}-\bar{RRI}|<1.5*Std(RRI\right)\\ 0.7*RRI_{i-1}<RRI_{i}<1.3*RRI_{i-1} \end{array}$$

where *RRI* is the *RR* intervals, *RRI_i_*_−1_ and *RRI_i_* are adjacent intervals.

After processing ECG and respiratory signals, we calculated CRI sequences taking the step size of 10 s with the window of 120 s.

### 2.2. Methods

#### 2.2.1. Cardiopulmonary Resonance Indices (CRI)

Cardiopulmonary interaction is important in the circulation system to ensure efficient delivery of oxygen and nutrients, and that the efficiency is optimized at the state of cardiopulmonary resonance. Cardiopulmonary resonance indices (CRI) come from the bivariate autoregressive model of respiration series and RR intervals. The model could calculate respiratory along with the non-respiratory component effects on RR intervals in the frequency domain based on *G*-causality [42].

In the model, the change process of *RRI* was regarded as a Markov process, ignoring other factors affecting heart rate in the short term, the *RRI* and *RSP* (both of length T) were described by a bivariate auto-regressive model [43]:

(3) $$\begin{array}{l} RRI(t)=\sum_{j=1}^{p}A_{11,j}RRI(t-j)+\sum_{j=1}^{p}A_{12,j}RSP(t-j)+\varepsilon_{1}(t)\\ RSP(t)=\sum_{j=1}^{p}A_{21,j}RRI(t-j)+\sum_{j=1}^{p}A_{22,j}RSP(t-j)+\varepsilon_{2}(t) \end{array}$$

where *p* is the maximum number of lagged observations included in the model (the model order, *p* < T). *A* contains the coefficients of the model, and *ε*_1_, *ε*_2_ are the residuals for each time series.

By the definition of *G*-causality, the magnitude of RSA can be measured by the log ratio of the prediction error variances for the restricted (*R*) and unrestricted (*U*) models:

(4) $$G_{2\to 1}=\ln\frac{\mathrm{var}(\varepsilon_{1R(12)})}{\mathrm{var}(\varepsilon_{1U})}$$

where ε_1*R*(12)_ is derived from the model omitting the *A*_12,*j*_ (for all *j*) coefficients in the first equation and ε_1*U*_ is derived from the full model [21]. In order to describe the effect of respiration on heart rate better, and to compare the model with Heart Rate Variability (HRV), the model conducted the operation above in the frequency domain. Thus, through the model, we obtained a curve *G*(f) which measures the cardiopulmonary interaction in the frequency domain.

**Figure 2:**
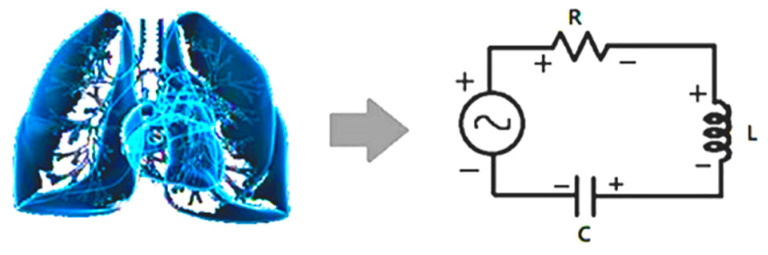
Schematic diagram of cardiopulmonary system and its circuit model. Interaction between lung and heart resembles the energy flow between inductor and capacitor. The non-respiration factors are equivalent to resistor, damping the resonance.

Based on *G*(f) in the frequency domain, CRI are defined to measure the status of the cardiopulmonary resonance system by adopting the cardiopulmonary system circuit model [42,55,56] (Figure 2). 

**Figure 3:**
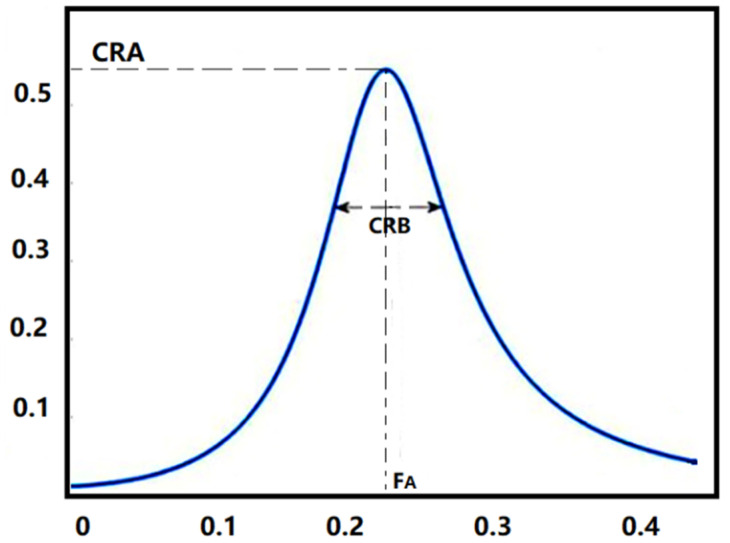
Cardiopulmonary Resonance Indices (CRI). CRA is the maximum amplitude of the curve, and CRB is the bandwidth of the curve. *F_A_* is the cardiopulmonary resonance frequency.

CRI include cardiopulmonary resonance amplitude (CRA), cardiopulmonary resonance bandwidth (CRB) and cardiopulmonary resonance quality factor (CRQ) shown in Figure 3. CRA represents the coupling depth of the cardiopulmonary system, CRB represents the width of the coupling frequency band, and *CRQ* represents the current quality of the cardiopulmonary system. CRI are the quantitative measurements for respiratory sinus arrhythmia (RSA) in the frequency domain [42]. Reflecting the modulation effect of breathing on heart rate changes, CRI well represent the degree of cardiopulmonary resonance and parasympathetic nerve activity level. They have been proved valid in the classification of NON-REM (NREM) and REM sleep [42] and in the antepartum autonomic nervous care in pregnant women [43].

CRQ is defined to measure the merit of the cardiopulmonary resonance system by adopting the quality factor measure for inductor, capacitor, and resistor LCR oscillator. Combined with the physiological significance of the cardiopulmonary coupling model, we expanded the index system with non-respiration factors, which is an analogy with the energy consumption element in the circuit model, as cardiopulmonary resonance resistance (CRR).

In the oscillating system, the quality factor of a circuit is the ratio between the reactance power and the average power consumed by the resistor, namely the ratio of reactive power and active power. In the non-radiative system, the capacitive reactance is equal to the inductive reactance at resonance, then we can deduce [57]:

(5) $$\mathrm{CRQ}=\frac{1}{2\pi F_{A}CR}$$

where *F_A_* represents the cardiopulmonary resonance frequency. Then we define the cardiopulmonary resonance resistance (CRR) as follows [57]:

(6) $$\mathrm{CRR}=2\pi F_{A}CR$$

Cardiopulmonary resonance resistance (CRR) represents the physiological phenomena of people that exist in the heart rate variability excluding the respiratory modulation, and is related to pathological state of the subject.

CRA, CRB, CRQ and CRR jointly constructed the evaluation index system of cardiopulmonary coupling. In the following analysis, we constructed the sleep analysis scheme by using the performance of the four indicators (including CRQ, CRR, CRB and CRA) in different groups. The significant difference of the CRI in A-phase and non-A phase (NA-phase) period were detected by the repeated one-way ANOVA test.

#### 2.2.2. CAP Recognition and Disease Diagnostic Scheme

Inspired by the results of the statistical analysis of the cardiopulmonary characteristics of healthy people and patients with sleep-related diseases, we propose an automatic recognition and disease diagnostic scheme of CAP based on CRI. Using the Hidden Markov and Random Forest model, the scheme combines the values and stability of characteristics which measure the status of cardiopulmonary system of people during sleep. Precision and recall rates will be calculated in the next section.

**Figure 4:**
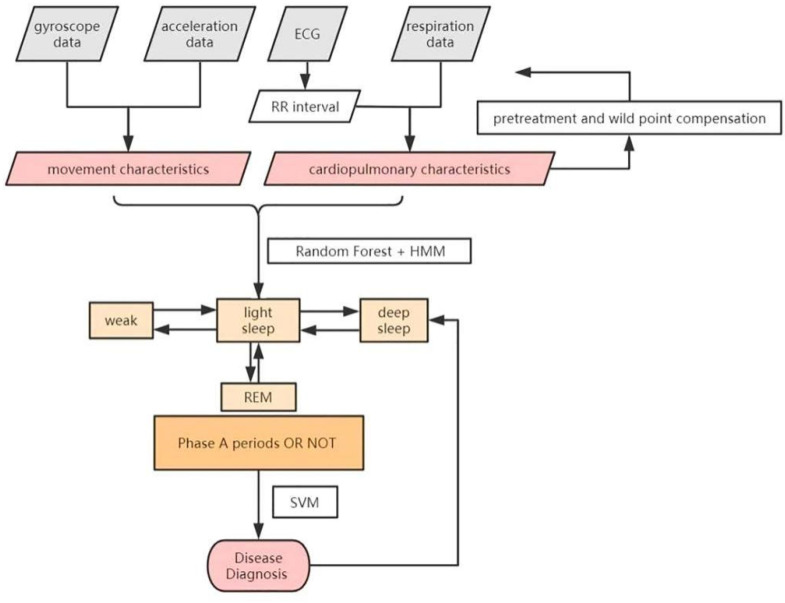
The classification and diagnosis scheme.

The flow of the classification and diagnosis scheme is shown in the Figure 4 above. In our work, movement characteristics and cardiopulmonary characteristics are extracted every 30 s, along with improved Hidden Markov and Random Forest model, to classify the sleep stages. Support Vector Machine (SVM) is then used to diagnose diseases.

The classification algorithm in this paper uses a total of 21 features, consisting of three body movement (BM) features, 12 heart rate variability (HRV) features and six CRI features. Each kind of data is preprocessed.

Body movement (BM) features [58] include the variance, approximate entropy and sample entropy of three cycle acceleration signals. HRV features used in this paper can be split into three categories following existing research [59]. Three time-domain features: SDNN, RMSSD, pNN50. Four frequency-domain features: VLF power, LF power, HF power, LF/HF ratio. Five nonlinear features: SD1, SD2 and SD1/SD2 from Poincare plot, as well as sample entropy and approximate entropy. CRI features include follows: CRR, CRB, CRQ, CRA, the variance of CRA, the entropy of CRA. 

During the training process, to improve the effectiveness of the extracted features, for the sleep stage WEAK, LIGHT SLEEP (stages S1 and S2) and DEEP SLEEP (stages S3 and S4), 20 consecutive tagged data is selected as the training data, that is, the data remaining unchanged in sleep status within 10 min is selected as the initial training data. For the identification of A1-phase, A2-phase and A3-phase, all the data are used. When the value of the calculated feature exceeds the normal range, the data fragment is preprocessed again, and the outliers are compensated until the eigenvalue is normal. 

The scheme adopts the combination of Hidden Markov and Random Forest model to fully utilize the variation and stability of features.

The Hidden Markov Model is used to characterize both the stability and the variation of CRA between different sleep stages, and the final recognition scheme is realized by the Random Forest Model. 

The Hidden Markov Model is a probabilistic model of timing sequences. It can learn the implicit characteristics of states by observing phenomena. The transformation process between sleep stage is a Markov process with implicit temporal information. The physiological signals and sleep states can be observed, while the numerical characteristics of physiological significance of the state transitions are unclear. Therefore, we choose the hidden Markov model as the initial model. In this study, for each subject, the transformation between the sleep stages and phases is a Markov chain. 

We take the CRA sequence as the observation sequence and the label of the sleep stages as the state sequence. There are 18 kinds of sleep stages in this study, including WEAK, S1 (A1, A2, A3 and NA), S2 (A1, A2, A3 and NA), S3 (A1, A2, A3 and NA), S4 (A1, A2, A3 and NA) and REM. *I* is the state sequence of 18 dimension, and *O* is the corresponding observation sequence of CRA.

(7) $$I=\left(i_{1},i_{2},\ldots ,i_{18}\right)$$

*A* is the probability matrix of the observation transition:

(8) $$A={\left[a_{ij}\right]}_{18\times 18}$$

(9) $$a_{ij}=P(O_{t+1}=q_{j}|O_{t}=q_{i}),i=1,2,\ldots ,18;j=1,2,\ldots ,N$$

where *a_ij_* is the probability of the transformation to *q_j_* at time *t* + 1, given *q_i_* at time *t*.

In the CAP classification scheme, firstly, we train the HMM [44] for CRA and corresponding sleep stage tags. Then, the values along the diagonal of A and the sum of each column are calculated as the new eigenvalues of the states of the corresponding columns to represent the stability of CRA. Finally, we put the two new features and the 21 features mentioned earlier as the inputs of the Random Forest Model. 

The Random Forest Model has natural advantages for classification tasks with many features and incomplete sample size, with high accuracy and fast training speed. It is suitable as our final classifier. The training and classification work are carried out in two layers, the first layer is carried out for identification stage WEAK, stages REM, S1, S2, S3 and stage S4, while the second layer is carried out for identifying A1-phase, A2-phase and A3-phase in S1/S2/S3/S4, respectively. The clarification of the two layers both used Random Forest Model. 

SVM [60] is used in the scheme to diagnose insomnia and narcolepsy. The output of the Random Forest Model and the 23 characteristics above are used for training of the SVM. For subjects diagnosed with sleep-related disorders, their eigenvalues will be recalculated and retained in the training library. 

## 3. Results

### 3.1. Results of the Statistical Analysis of CRI in People with Non-Pathology, Insomnia and Narcolepsy

This section describes the statistical analysis of the cardiopulmonary characteristics of people with non-pathology and several sleep-related pathologies.

The deep sleep stage period (including stages S3 and S4) during the whole sleep, in which the human body is least affected by external noise and voluntary activities, shows more remarkable physiological significance in analyzing statistical results [61]. Previous studies have shown that CRI characteristics are stable and have statistical significance during deep sleep stage [43]. 

**Figure 5:**
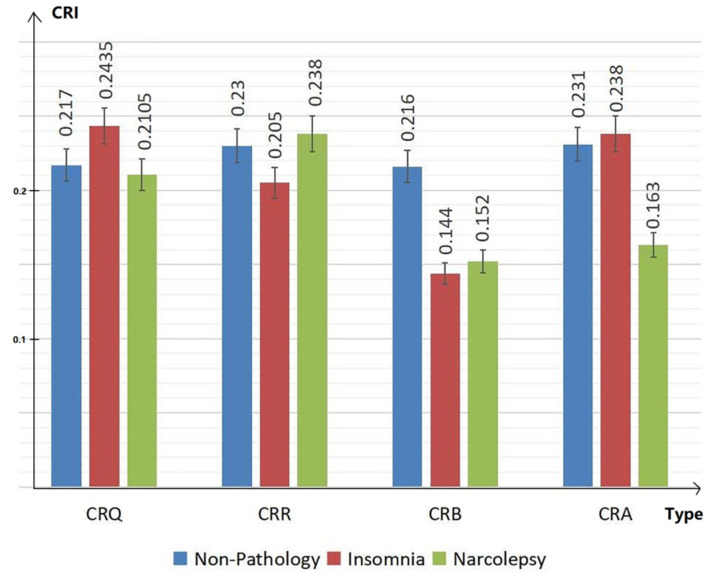
Cardiopulmonary characteristics CRQ, CRR, CRB and CRA during deep sleep of people with non-pathology, insomnia and narcolepsy.

Therefore, CRI characteristics (including CRQ, CRR, CRB and CRA) in people with non-pathology, insomnia and narcolepsy during the DEEP SLEEP period were analyzed. The results are shown in the Figure 5 below. The values of CRI are normalized in the figure for convenience of comparison. The blue bar shows the results of group with non-pathology. The red bar shows the results of group with insomnia. The green represents the results in subjects with narcolepsy. 

In the bar chart Figure 5, we can see that, compared to people with non-pathology, insomniacs have higher CRQ and lower CRR, while narcolepsy patients show the opposite manifestations. The CRB of the healthy group is significantly higher than the CRB of insomniacs and narcolepsy. At the same time, the CRA of insomniac patients is higher, while the CRA of narcolepsy patients is significantly lower than the CRA of the healthy group. The overall coupling of depth and width of narcolepsy patients are low.

For the analysis of CAP mode, the repeated one-way ANOVA, followed by Dunnett’s post hoc test [62] is used to represent the significant difference of CRA in the A-phase and non-A phase (NA-phase) period during the sleep stages S3 and S4 in Table 1 below as an example.

It is a method of comparing means in analysis of variance to judge whether the influence of CRA on the sleep stage is significant [62]. For repeated one-way ANOVA, if *p* < 0.05, there is significant difference between the groups. We calculated the *p* value of CRA in S3 and S4: *p* = 0.004. For Dunnett’s post hoc test, if the value of difference of the mean > LSR, there is a significant difference between the groups being compared. 

In the Table 1, it can be seen that CRA, as a measure of coupling depth, has significant differences between A-phase and NA-phase. For the different groups, measurements of people with non-pathology, insomnia and narcolepsy were analyzed and compared to each other below.

**Figure 6:**
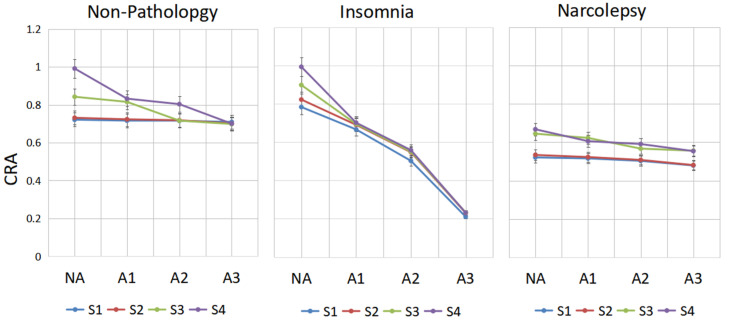
The change line diagram of CRA of different people in CAP. CRA in period A1-phase, A2-phase, A3-phase and NA-phase in sleep stages S1, S2, S3 and S4 are shown for every group.

In the groups with non-pathology, insomnia and narcolepsy, by comparing the performance of CRA in period A1-phase, A2-phase and A3-phase in sleep stages S1, S2, S3 and S4 shown in Figure 6, the following results are obtained.

In the non-pathology group, in stage S4, there is a significant drop in CRA from A2 to A3; in stage S3, there is a huge drop between A1 and A2, whereas S1 and S2 are inherently less coupled. CRA of A3 in stages S3 and S4 is lower than in stages S1 and S2 in the NA-phase. In insomniacs, CRA of A3-phase in stage S3 is very low, while it is very high in the NA-stage. We can see that CRA fluctuates greatly between phase A and non-Phase A. This may be a compensatory mechanism of the autonomic nervous system in insomniacs. In narcolepsy, the CRA in S3 and S4 is small regardless of whether they are in an A-phase or NA-phase period.

**Figure 7:**
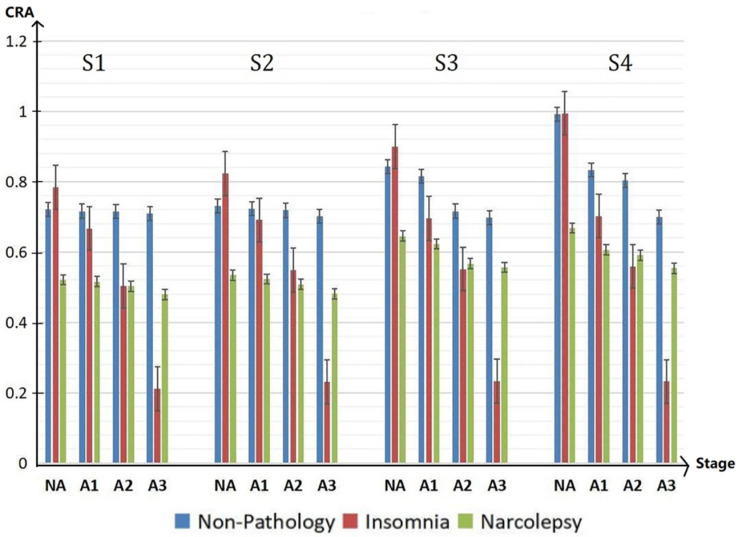
CRA in NA, A1, A2 and A3 period of different groups in the sleep stages S1, S2, S3, and S4 during the whole sleep.

To facilitate the analysis and comparison between three groups, Figure 7 is drawn. From Figure 7, compared with the healthy group, the cardiopulmonary performance of insomniacs fluctuates greatly with the generation of A-phase. The value of CRA is very large in the non-A phase period and very small in the A-phase period. However, the value of CRA of narcolepsy patients is generally small. Combined with the overall analysis of CRB and CRR, it can be concluded that the cardiopulmonary coupling damping of narcolepsy patients is relatively high.

Overall, from the detailed analysis of the cardiopulmonary characteristics of different groups during the sleep period, we could see that CRI characteristics (including CRQ, CRR, CRB and CRA) in people with non-pathology, insomnia and narcolepsy are different during the whole sleep. The CRI could help diagnose insomnia and narcolepsy. In the CAP mode of different groups, both the variation and stability of CRA characteristics are significantly different. Constructing a classifier combining variation and stability can help us to recognize CAP more accurately.

### 3.2. Results of the Recognition and Disease Diagnostic Scheme

To illustrate the classification performance and generalization ability of the scheme, we conducted the 7-fold cross-validation [63]. We proved the validity of the sleep stage classification method by comparing our estimates with the expert diagnosis results.

The sleep stage classification results are shown in the confusion matrix Table 2. Each value in the table represents the number of samples. Each column represents the prediction category. Each row represents the actual category the data belong to. The results include 18 categories: Wake, REM, S1, S2 and S3, S4. In the S1, S2, S3 and S4, there are A1, A2, A3 and NA in every stage. According to Table 2, the accuracy and recall rates of the different classes could be calculated. 

In order to express classification accuracy and generalization performance form Table 2, the F1 score is defined as the average of accuracy and recall rates of all the eighteen classes [64].

(10) $$F1_{i}=2*\frac{precision_{i}*recall_{i}}{precision_{i}+recall_{i}}$$

In this formula, *i* stands for class *i*, including WEAK, REM and A1, A2, A3 and NA from stage 1 to stage 4. The precision refers to the specific gravity of the positive sample in the positive example determined by the classifier; recall refers to the proportion of the total positive cases that are predicted to be positive. Then the final F1 score in CAP experiments is [64]:

(11) $$F1=\frac{\sum_{i=1}^{18}F1_{i}}{18}$$

According to the Table 2, we could calculate that F1 score of sleep-wake classification is 92.0%. F1 score in the sleep stage classification (WEAK, REM, S1, S2, S3 and S4) is 83.8%, while the F1 score in the CAP experiments is 80.4%. 

In detail, about the identification of A-phase, the accuracy in stages S1, S2, S3 and S4 are 84.4%, 90.1%, 84.2% and 79.9% respectively. The average recognition rate of A-phase reaches 84.7%. Similarly, in terms of disease diagnosis, the F1 score of diagnosis and recognition is 87.8% for insomnia and 90.0% for narcolepsy. For real cases, this table could help to analysis differences between the results of sleep classification and the actual sleep structure easily.

## 4. Discussion

CRI characteristics (including CRQ, CRR, CRB and CRA) in people with non-pathology, insomnia and narcolepsy are different in all stages during the whole sleep. 

Compared to people with non-pathology, insomniacs show higher CRQ and lower CRR, while narcolepsy patients have the opposite manifestations. The CRB of the healthy group is significantly higher than the CRB of insomniacs and narcolepsy. At the same time, the CRA of insomniac patients is higher than the healthy group, while the CRA of narcolepsy patients is significantly lower than that of the healthy group. This phenomenon can be considered as a compensation of autonomic nerve of insomniac patients. Meanwhile, the coupling depth CRA and coupling width CRB of narcolepsy patients are always low during the whole sleep. 

In the CAP, in stage S4, there is a significant drop in CRA from A2 to A3; in stage S3, there is a sharp drop between A1 and A2, whereas S1 and S2 are inherently less coupled. CRA of A3 in stages S3 and S4 is lower than in stages S1 and S2 in the NA-phase. These performances support the conclusions including the presence of heart-brain connection [39] and the EEG performance of A2 and A3 type [50]. Compared to people with non-pathology, the cardiopulmonary performance of insomniacs fluctuates greatly with the generation of A-phase. The CRA is very large in the non-A phase period and very small in the A-phase period. However, the CRA of narcolepsy patients is generally low. Combined with the analysis of CRB and CRR, it can be concluded that the cardiopulmonary coupling damping of narcolepsy patients is relatively high. 

In the CAP mode of different groups, both the variation and stability of CRA are significantly different. Our scheme using improved HMM and RF combines these two points well. The average recognition rate of A-phase reaches 84.7% and the F1 score in the CAP experiments reaches 80.4%. 

In order to illustrate the role of CRI features in the scheme, as the control group, the features related to CRI were removed while the remaining features were used. After the training of the Random Forest Model and 7-fold cross-validation test, the F1 score of CAP classification is 73.8%. The F1 score of disease diagnosis is 71.6%. Besides, as comparison, features in references [34,35,36,37,38] are used in our scheme instead of CRI. Meanwhile, research on the feature extraction methods of ECG in recent ten years [65,66,67,68] are also considered. The results are shown in Table 3. 

It can be seen that the performance of the CRI surpasses that of the features in other studies in sleep stage classification, especially in CAP pattern recognition. In the diagnosis of sleep-related disorders, CRI significantly outperforms other features.

The performances of ‘removing CRI’ and ‘CRI’ are better than the other benchmark papers, such as [34,35,36,37,38]. These papers start with the heart rate and body movement signals, try to find features that can characterize sleep stages. The HRV features in our classifying framework, include: three time-domain features: SDNN, RMSSD, pNN50; four frequency-domain features: VLF power, LF power, HF power, LF/HF ratio; five nonlinear features: SD1, SD2, SD1/SD2, sample entropy and approximate entropy. Body movement features include the variance, approximate entropy and sample entropy of three cycle acceleration signals. These are more comprehensive than those in these papers. ‘CRI’ performances better than [67,68], which uses wavelet filter bank to analysis the heart rate characteristics. The wavelet analysis could dig out heart rate characteristics in depth, which are closely related to the activities of cardiopulmonary system, to some extent. Compared with the features of the directly acquired signals, such as ECG, three-axis acceleration signals, etc., CRI deeply explores the degree of cardiopulmonary coupling in different stages of sleep, shows the physiological significance of the autonomic nerve regulation. 

CRI characteristics quantify the coupling status in the cardiopulmonary system, and could express the activity characteristics of autonomic nerve regulation. These autonomic nerve regulation characteristics are determined by EEG, and ultimately manifest as tiny periodic changes in sleep stages. At the same time, using Hidden Markov and Random Forest model, the scheme proposed combines the values and stability of these characteristics during sleep. This makes the results of classification more accurate. For the groups with non-pathology, insomnia and narcolepsy, CRI show significant differences in various stages of sleep and express the pathological features of the disease. Thus CRI make the framework more effective in diagnosing diseases. 

The results of the scheme, on the one hand, prove the existence of a heart-brain connection, and on the other hand, verify the validity of CRI in the expression of the cardiopulmonary coupling and autonomic nervous activity of human being. The scheme is also helpful to identify insomniac patients and narcolepsy patients. 

## 5. Conclusions

Cyclic Alternating Pattern (CAP) is a sensitive tool for studying sleep disorders throughout the life cycle. Several efforts have been made to develop a reliable automatic CAP-scoring algorithm. Most of these methods rely on the extraction of spectral features from the EEG.

Considering the connection between the heart and brain of people, we analyze the cardiopulmonary characteristics of cardiopulmonary resonance indices (CRI) in people with non-pathology, insomnia and narcolepsy under the CAP mode. We analyze and identify key fields that contribute to insomnia and narcolepsy. The results show that CRI have different manifestations in different groups. The conclusion is drawn that CRI are capable of representing the cardiopulmonary coupling degree, autonomic nerve state and sensitivity of the subjects, and thus are able to measure the health status of human body.

Inspired by the results of the statistical analysis of the cardiopulmonary characteristics, our CAP Recognition and Disease Diagnostic scheme uses CRI in the frequency domain as the feature to recognize the A-phase stage during the whole sleep.

There are many trials in pattern recognition using Hidden Markov and Random Forest model. Different methods have shown superiority in different applications. The scheme in this article takes both variation and stability of measurements of coupling state of the cardiopulmonary system during sleep into account. The precision and recall show the good performance of the scheme.

The proposed method is effective to conquer the weakness about the signal acquisition and processing in clinical trials, particularly when we consider the stress of the examinee and the cost of measurements using the conventional Rechtschaffen & Kales rules [50]. Results show that the scheme could automatically recognize the Cyclic Alternating Pattern accurately, and hence help to diagnose insomnia and narcolepsy. Further, we would conduct more clinical trials to validate and improve our model.

To develop a classification and diagnosis scheme for a specific hospital, the clinical data and expert opinions should be added into the scheme. It could be interesting to see how these contribute to the prediction accuracy.

Furthermore, the meaning and application of CRI are also worth exploring, such as the psychological stress, inflammation, etc.

## Figures and Tables

**Table 1:** Repeated one-way ANOVA and Dunnett’s post hoc test. If the value of difference of the mean > LSR, there is a significant difference between the groups being compared (*p* = 0.004 < 0.05).

CRA in Deep Sleep	S3		S4	
	Difference of the Mean	LSR (*p* < 0.05)	Difference of the Mean	LSR (*p* < 0.05)
A and NA	0.134	0.047	0.216	0.096

**Table 2:** The confusion matrix of the sleep stage classification.

		Pre		S1				S2				S3				S4			
		W	R	NA	A1	A2	A3	NA	A1	A2	A3	NA	A1	A2	A3	NA	A1	A2	A3
Act	W	3622	102	57	48	72	316	112	70	58	49	2	3	3	22	3	1	4	20
	R	70	824	20	28	45	40	32	23	19	26	1	3	3	12	2	1	2	4
S1	NA	52	16	2789	138	164	119	293	3	2	2	1	1	14	18	1	1	13	30
	A1	73	28	5	383	81	48	3	4	7	21	1	1	1	3	1	1	1	3
	A2	79	30	18	68	403	39	2	2	4	24	0	1	1	3	0	0	1	4
	A3	86	30	6	13	17	309	1	1	4	27	0	0	1	4	0	0	0	5
S2	NA	55	26	250	9	3	1	2894	87	64	56	2	13	10	4	2	4	8	14
	A1	52	23	27	4	2	0	20	243	42	16	0	2	3	2	1	1	2	2
	A2	49	20	17	10	3	1	6	32	200	26	1	1	3	2	1	1	3	3
	A3	64	33	14	15	2	1	8	17	21	268	1	1	1	3	0	1	1	5
S3	NA	3	0	8	1	0	0	6	2	0	0	161	14	8	1	19	1	1	0
	A1	2	2	3	1	0	0	1	1	1	0	4	46	5	2	7	1	0	0
	A2	1	0	2	1	0	0	1	1	1	0	2	4	31	3	6	1	0	0
	A3	1	4	1	1	1	0	1	2	0	0	1	2	3	33	6	1	1	1
S4	NA	2	1	7	0	0	0	15	1	0	0	19	2	0	0	151	11	10	5
	A1	1	1	3	0	0	0	3	0	0	0	6	1	1	0	2	44	3	1
	A2	1	1	2	1	0	0	1	1	0	0	2	3	1	1	2	4	31	2
	A3	1	3	1	1	0	0	1	1	0	0	2	3	2	2	1	1	4	37

The value of row i and column j represents the number of the sections whose truth value is class i and is predicted to be class j. Each column represents the prediction category; Each row represents the actual category of data belonging to. ‘W’ represents Wake, ‘R’ represents REM. ‘Act’ represents the number of actual sleep stage; ‘Pre’ represents the number of the predicted sleep stage.

**Table 3:** The F1 scores of CRI and features of other studies in experiments of sleep stage classification and disease diagnosis.

Method	Sleep-Wake Classification	S1, S2, S3, S4 and Wake Stage Classification	CAP Recognition	Disease Diagnosis
Heart rate spectrum analysis [34,65]	77.6%	72.6%	66.7%	70.5%
detrended fluctuation analysis [35]	78.6%	71.4%	66.3%	64.7%
time-varying spectral features [36,37]	82.0%	76.6%	70.3%	72.5%
Heart rate fluctuations [38,66]	79.9%	73.1%	66.7%	70.5%
wavelet filter bank [67,68]	90.1%	82.6%	76.7%	80.9%
Removing CRI	85.9%	77.7%	73.8%	71.6%
CRI	92.0%	83.8%	80.4%	88.9%

## Data Availability

The data for this article is publicly available on the website https://www.physionet.org/content/capslpdb (accessed on 1 January 2022).
